# Correction to: Prenatal and Postnatal Cardiac Development in Offspring of Hypertensive Pregnancies

**DOI:** 10.1161/JAHA.119.014640

**Published:** 2021-01-13

In the article by Aye et al, “Prenatal and Postnatal Cardiac Development in Offspring of Hypertensive Pregnancies,” which published online April 30, 2020, and appeared in the May 5, 2020 issue of the journal (*J Am Heart Assoc.* 2020;9:e014586. DOI: 10.1161/JAHA.119.014586), a correction was needed.

The incorrect publication agreement was selected prior to publication. The article was published under a Creative Commons Attribution‐NonCommercial‐NoDerivs License, but to comply with a funding mandate, should have been published under the terms of the Creative Commons Attribution License. The license has now been corrected and the statement now reads: “© 2020 The Authors. Published on behalf of the American Heart Association, Inc., by Wiley. This is an open access article under the terms of the Creative Commons Attribution License, which permits use, distribution and reproduction in any medium, provided the original work is properly cited.”

The authors regret the error.

The online version of the article has been updated and is available at https://www.ahajournals.org/doi/10.1161/JAHA.119.014586

